# Tuberculous sarcoidosis: Is it a separate entity?

**DOI:** 10.4103/0970-2113.53225

**Published:** 2009

**Authors:** Ritesh Agarwal, Dheeraj Gupta

**Affiliations:** *Department of Pulmonary Medicine, Postgraduate Institute of Medical Education and Research, Chandigarh - 160 012, India. E-mail: riteshpgi@gmail.com/ritesh@indiachest.org*

Relationship of tuberculosis (TB) with sarcoidosis has baffled physicians and researchers ever since the description of sarcoidosis. Not only sarcoidosis is a close clinical mimic of TB, which makes the diagnosis of sarcoidosis (*vs*. TB) difficult in countries with high prevalence of TB, and *vice versa*, but TB has also been linked to causation of sarcoidosis. All those who deal with such conditions would remember the cases, when TB, after successful treatment (or during it), is followed by a diagnosis of sarcoidosis and more commonly when patient being treated with steroids following with a diagnosis of sarcoidosis, land up with TB. In both of the clinical scenarios, the authenticity of the primary diagnosis has to be confirmed, before one can say that either of the conditions has lead to the other one.

In this issue of the journal, Shah *et al*. present an interesting viewpoint regarding an entity called ‘tuberculous sarcoidosis’. Further, the authors have even laid down the criteria for the diagnosis of tuberculous sarcoidosis.[1] Tuberculous sarcoidosis was a term given to the patients who had features of both TB and sarcoidosis, way back in 1960.[2] However, many things have changed since then and good knowledge about the histopathological variations is observed in TB and sarcoidosis has evolved. So is there any merit in reviving this old forgotten term?

For example, in the illustrative case 1 described by Shah *et al*. [originally by Wong *et al*.,[3] represents a typical presentation of sarcoidosis coexisting/following a case of bacteriologically confirmed TB, and there is no question of labeling the case as tuberculous sarcoidosis. Similarly, the case 2,[4] could be a presentation of sarcoidosis as pleural effusion with spontaneous resolution of pleural effusion[5] followed by development of typical sarcoid manifestations or could be sarcoidosis following a tuberculous pleural effusion. Cases 3 and 4 are again examples of coexisting TB and sarcoidosis with sequential and uninterrupted progression of one disease into another. Case 5 is the most typical presentation of sarcoidosis which one can encounter and just on the basis of fibrinoid necrosis resembling caseation the patient was initiated on antituberculous therapy.[6] The illustrative cases reveal only one fact: That is, the clinician should be astute to pick up the typical presentations. Finally, the diagnostic criteria laid down by Shah *et al.* [Table 1] are highly nonspecific and the only specific findings are those that are well known to be associated with sarcoidosis, and include typical clinical presentation with characteristic radiological and histopathological findings.

**Table 1:** Diagnostic criteria proposed by Shah *et al*.

Criteria	Diagnostic value
Age: 25-45 years.	Nonspecific
Gender: No gender predisposition.	Nonspecific
History of previous TB infection which was adequately treated with antitubercular therapy with adequate drug combinations, dosages and duration	Nonspecific as history of antitubercular therapy is elicited in almost 40% of patients with or without previous documented TB
History of close contact with patient having TB infection or family history of TB or association with Type II diabetes mellitus	Nonspecific, association with type II diabetes mellitus of no proven evidence
Onset: Asymptomatic or with mild fever, anorexia and loss of weight	Non-specific
Important symptoms: chronic dry nonproductive cough, breathlessness on exertion	Nonspecific
Important signs: Involvement of multiple nodes for example, scalene or cervical groups oflymph nodes, at multiple locations, bibasilar end inspiratory velcro crackles on auscultation of chest	Nonspecific
Raised inflammatory markers—ESR	Nonspecific
Increased SACE level	Can be elevated in both conditions
Tuberculin test—Positive or negative	Nonspecific
At times patient may develop localized granulomatous reaction at the site of tuberculin test	Nonspecific
Sputum—Positive or negative for *M. tuberculosis*	False-positive acid fast bacilli or coexisting TB with sarcoidosis. Patient should never receive glucocorticoids alone
Culture—Negative for *M. tuberculosis*	
PCR of biopsy tissues is positive for *M. tuberculosis*	Seen in both TB and sarcoidosis
Biopsy of involved site shows noncaseating granulomas with classical confluence at times showing caseation necrosis	Typical of sarcoidosis
X-ray chest: Bilateral symmetrical hilar and right paratracheal lymphadenopathy	Typical of sarcoidosis
Pulmonary parenchymal infiltration which is usually bilaterally symmetrical involving themid-zone and upper-zone and absence of cavitations	Insensitive more likely to occur in sarcoidosis
CT scan of chest: micro and macro nodules which have characteristic distribution in peribronchovascular region, subpleural interstitium and interlobular septa	Typical of sarcoidosis
No expected response to antitubercular therapy	Insensitive more likely to occur in sarcoidosis
Dramatic response to steroids with resolution of symptoms	Insensitive more likely to occur in sarcoidosis

Since sarcoidosis was first described, there has always been a belief that the disease is in some way related to TB.[7] Sarcoidosis is known to follow or even coexist with TB,[2,8] and TB following sarcoidosis is a fairly common event due to the immunosuppressive effects of glucocorticoids.[9] Thus, it is no surprise that a physician is likely to encounter situations wherein sarcoidosis may precede, follow or present concurrently with TB. Rather than coining a separate term, it would be better to recognize the typical and atypical clinical presentation of sarcoidosis and diagnose it appropriately.[10,11]

Of late, nucleic acid amplification of the genetic material of putative agents serves as an alternative method for isolating fastidious organisms. This method has been used successfully in identifying *Tropheryma whippelli* as a causative agent for Whipple's disease[12] and coronaviruses[13] as an agent for severe acute respiratory syndrome. In a recent meta-analysis, we have shown that there is a 25.6% (95% CI, 23.6 – 29.5%) prevalence of mycobacterial DNA in patients with sarcoidosis with the odds of finding mycobacteria in samples of patients with sarcoidosis versus controls being 9.67 (95% CI, 4.56 – 20.5).[14] These results indicate that there is a definite link between TB and sarcoidosis. It might be appropriate to consider that the situation of sarcoidosis is analogous to leprosy in which the tuberculoid (pauci-bacillary) and lepromatous (multi-bacillary) forms are encountered; and, sarcoidosis might represent the tuberculoid form of the pathological responses to mycobacteria.[15]

Based on the current understanding, we believe that there is no merit in the term “tuberculous sarcoidosis”; and the criteria laid down by Shah *et al*. are neither evidence-based nor play any role in day-to-day clinical practice.
